# IFN‐γ‐Associated Signaling Networks in Interstitial Lung Disease Associated With Sjögren's Disease

**DOI:** 10.1002/iid3.70547

**Published:** 2026-10-06

**Authors:** Qianqian Chen, Yuhan Li, Yiming Chen, Yuhua Su, Xiaodong Wang, Chunxiao Guan

**Affiliations:** ^1^ Department of Rheumatology and Immunology Shandong Province Rheumatic Disease Key Speciality Affiliated Hospital of Shandong Second Medical University Weifang China; ^2^ Key Laboratory of Rheumatology and Immunology Weifang China; ^3^ Department of Clinical Research Center Affiliated Hospital of Shandong Second Medical University Weifang China

## Abstract

**Background:**

Sjögren's disease (SD) is a chronic systemic autoimmune disorder that can affect multiple organ systems. Interstitial lung disease (ILD) is a clinically significant pulmonary manifestation of SD and is associated with impaired quality of life and increased mortality. Interferon‐γ (IFN‐γ), a major effector cytokine of Th1 immunity, has been implicated in immune dysregulation and chronic inflammation; however, its contribution to SD‐associated ILD (SD‐ILD) and its relationship with fibrotic remodeling remain incompletely understood.

**Materials and Methods:**

This pathway‐oriented narrative review synthesized evidence identified through a structured and iterative search of PubMed, Embase, and Web of Science, updated through August 2026. Priority was given to direct evidence from SD‐ILD. When disease‐specific evidence was limited, mechanistic findings from SD without characterized ILD, related connective tissue disease‐associated ILDs, fibrotic lung diseases, and experimental models were considered and interpreted as indirect or extrapolated evidence.

**Results:**

Available evidence supports a conceptual network in which IFN‐γ may function as a potential signaling node interacting with JAK/STAT, PI3K/AKT, NF‐κB, MAPK, WNT/β‐catenin, and IDO–kynurenine–AhR pathways. These pathways may contribute at different levels to immune‐cell activation, inflammatory amplification, immunometabolic regulation, epithelial injury, fibroblast activation, and fibrotic remodeling. Evidence is strongest for the biological relevance of IFN‐γ–JAK/STAT signaling, whereas support for PI3K/AKT, NF‐κB, and MAPK is more indirect, and the relevance of WNT/β‐catenin and particularly IDO–Kyn–AhR to SD‐ILD remains less well established.

**Discussion:**

The proposed IFN‐γ‐associated network should be regarded as a conceptual mechanistic framework rather than a fully validated signaling hierarchy in SD‐ILD. Distinguishing direct disease‐specific evidence from mechanistic extrapolation highlights important knowledge gaps and provides a rationale for future investigation of pathway‐informed biomarkers and stage‐specific therapeutic strategies. Prospective SD‐ILD studies integrating longitudinal clinical data with spatial and single‐cell approaches are needed to validate these relationships and clarify their translational relevance.

## 1 Introduction

Sjögren's disease (SD) is a chronic, systemic autoimmune disorder that principally targets the exocrine glands. Its hallmark clinical features—xerostomia and keratoconjunctivitis sicca—arise from progressive dysfunction of the salivary and lacrimal glands, respectively [1]. Beyond exocrine‐gland involvement, SD can affect the lungs, kidneys, liver, and nervous system, underscoring its systemic nature. Pulmonary manifestations, in particular, are recognized as major determinants of morbidity and mortality in these patients [2].

Interstitial lung disease (ILD) is the most common pulmonary manifestation of SD, with epidemiologic studies placing its prevalence at 3%–11% [3]. Once ILD develops, quality of life declines sharply; mortality rises to 16% at 5 years, and the 10‐year risk of death is quadrupled [2, 4, 5]. Although SD‐ILD carries major clinical consequences, its pathogenesis remains only partially defined. A prevailing view holds that dysregulated immune‐cell activation, sustained chronic inflammation, and the aberrant expression of multiple inflammatory cytokines converge to fuel pulmonary inflammation and tissue injury; consistent with this concept, studies have documented the aberrant infiltration of T and B lymphocytes into the lung parenchyma [6, 7]. Sustained elevation of tumor necrosis factor‐α (TNF‐α), interleukins (ILs), and other pro‐inflammatory mediators may likewise play a pivotal role in the initiation and progression of SD‐ILD [8, 9].

Among the plethora of inflammatory mediators, interferon‐γ (IFN‐γ)—the cardinal effector cytokine of Th1 cells—plays a major role in the immune dysregulation and tissue injury that characterize diverse autoimmune disorders [10]. IFN‐γ is a pleiotropic cytokine that not only underpins antiviral and antitumor immunity but also shapes the trajectory of autoimmune disease by modulating both innate and adaptive immune responses [11]. In patients with SD, elevated circulating IFN‐γ has been reported and associated with higher disease activity [12]. IFN‐γ engages the JAK‐STAT axis to transcriptionally up‐regulate a broad repertoire of pro‐inflammatory genes, thereby fueling immune‐cell activation and tissue infiltration. Concomitantly, it cross‐talks with NF‐κB, MAPK, and other signaling networks to amplify and perpetuate the inflammatory response [10, 11, 12, 13]. Such immunologic and inflammatory derangements have been implicated in lung injury across broader pulmonary inflammatory settings [14].

Notably, the role of IFN‐γ in pulmonary fibrosis remains contentious. Early work suggested that the cytokine might exert anti‐fibrotic activity by curbing fibroblast proliferation and dampening collagen synthesis [15]. Emerging evidence suggests that, in certain chronic inflammatory settings, sustained IFN‐γ signaling may contribute indirectly to fibrotic progression by modifying inflammatory and tissue‐repair responses [16, 17].

Nevertheless, mechanistic insight into how IFN‐γ governs immune dysregulation, inflammatory escalation, and fibro‐proliferative remodeling in SD‐ILD remains sparse, and a pathway‐level synthesis is still lacking. Mapping the signaling networks potentially associated with IFN‐γ should therefore refine our understanding of disease pathophysiology and highlight stage‐ and pathway‐specific therapeutic opportunities for future investigation Table 1.

**Table 1:** Proposed IFN‐γ‐associated signaling pathways potentially linking inflammation to fibrotic progression in SD‐ILD.

Pathway	Core biological function	Role in SD‐ILD progression	Evidence source and level of relevance to SD‐ILD
JAK/STAT (JAK1/2–STAT1)	IFN‐γ downstream inflammatory signal transduction	May contribute to immune activation, chemokine production, and pulmonary inflammation	SD and CTD‐ILD evidence; relatively stronger mechanistic support
NF‐κB pathway	Regulation of inflammatory gene expression and immune‐cell activation	May contribute to inflammatory amplification and immune‐cell activation	SD‐related immune evidence and chronic inflammatory disease models
PI3K/AKT pathway	Immunometabolic regulation, cell survival, and signaling adaptation	May contribute to immune‐cell activation, metabolic reprogramming, and B‐cell‐related immune responses	Autoimmune disease and experimental mechanistic evidence
MAPK pathway (p38/JNK/ERK)	Integration of inflammatory stress and cellular responses	May contribute to inflammatory amplification, epithelial responses, and fibrotic remodeling	Fibrotic lung disease and chronic inflammatory models
WNT/β‐catenin pathway	Regulation of tissue remodeling and fibroblast responses	May contribute to fibroblast activation and extracellular‐matrix remodeling	SD glandular fibrosis and fibrotic lung disease evidence
IDO–Kyn–AhR axis	Regulation of immune tolerance and tryptophan metabolism	May influence immune regulation and immune–structural cell interactions relevant to fibrotic remodeling	Predominantly indirect mechanistic evidence from immune and fibrotic disease models

## 2 Literature Search Strategy

This article was designed as a pathway‐oriented narrative review rather than a systematic review. A structured and iterative literature search was conducted in PubMed, Embase, and Web of Science. The search primarily focused on studies published within the preceding 5 years, while earlier studies were also considered when they provided foundational mechanistic evidence or when more recent SD‐ILD‐specific evidence was limited. The search was updated through August 2026.

The literature search was performed in a stepwise manner. First, studies directly addressing interferon‐γ (IFN‐γ), related signaling pathways, or relevant molecular mediators in Sjögren's disease‐associated interstitial lung disease (SD‐ILD) were sought. Second, when direct evidence in SD‐ILD was unavailable or limited, studies investigating the same pathways or mediators in Sjögren's disease without specifically characterized ILD were considered. Third, to explore potentially relevant pulmonary mechanisms, evidence from other autoimmune disease‐associated interstitial lung diseases, connective tissue disease‐associated ILD, idiopathic pulmonary fibrosis, and related experimental models of chronic lung inflammation or fibrosis was examined.

The initial search combined terms related to IFN‐γ, Sjögren's disease, and interstitial lung disease, including “interferon‐gamma,” “IFN‐γ,” “Sjögren's disease,” “Sjögren syndrome,” “interstitial lung disease,” “ILD,” and “pulmonary fibrosis.” Additional pathway‐specific searches were subsequently performed using terms related to JAK/STAT, PI3K/AKT, NF‐κB, MAPK, WNT/β‐catenin, and IDO–kynurenine–AhR signaling, together with terms referring to Sjögren's disease, autoimmune disease, interstitial lung disease, pulmonary inflammation, and pulmonary fibrosis. Reference lists of relevant articles were also manually screened to identify additional publications.

Original research articles, reviews, and meta‐analyzes relevant to the proposed mechanistic framework were considered. Priority was given to studies providing direct evidence in SD‐ILD. Evidence derived from Sjögren's disease without ILD or from other autoimmune and fibrotic lung diseases was used only when SD‐ILD‐specific data were lacking and is interpreted as indirect or extrapolated evidence within the mechanistic framework presented here.

Because this was a narrative rather than a systematic review, study selection was conducted iteratively according to relevance to the mechanistic questions addressed in this review. Titles and abstracts were initially screened to identify potentially relevant studies, followed by full‐text assessment of articles considered informative for the proposed pathway framework. Particular emphasis was placed on mechanistic relevance, evidence directly related to SD‐ILD, and studies that could clarify the biological relationships among the signaling pathways discussed. Because the search and selection process was iterative and was not designed as a systematic review, no PRISMA‐based study‐selection flow or formal quantitative record of retrieved, excluded, and included studies was generated. The purpose of the search was to construct and critically evaluate a mechanistic framework rather than to provide an exhaustive systematic evidence synthesis.

## 3 Clinical and Pathological Landscape of SD‐ILD

In SD‐ILD, the pulmonary histopathological spectrum encompasses several patterns. Nonspecific interstitial pneumonia (NSIP) is the most prevalent pattern, characterized by uniform alveolar septal inflammation and variable fibrosis. Usual interstitial pneumonia (UIP) pattern, marked by heterogeneous fibrosis with honeycombing and fibroblast foci, is less common but portends a worse prognosis. Lymphocytic interstitial pneumonia (LIP) and organizing pneumonia (OP) are also observed, particularly in patients with prominent lymphoproliferative features. The distribution of these patterns may have implications for disease behavior and prognosis [18]. More recently, a large cohort study of 558 patients with SD‐ILD showed that a radiological UIP pattern was associated with impaired pulmonary function and increased mortality compared with non‐UIP patterns [19]. High‐resolution computed tomography (HRCT) typically reveals ground‐glass opacities, reticulation, and traction bronchiectasis in NSIP‐predominant disease, whereas basal and peripheral honeycombing suggests UIP morphology. Pulmonary function tests consistently demonstrate restrictive ventilatory defect with disproportionate reduction in diffusing capacity for carbon monoxide (DLCO), reflecting alveolar‐capillary membrane dysfunction. Progressive annual decline in forced vital capacity (FVC) and DLCO identifies patients at heightened risk for respiratory failure [20]. Beyond systemic immune dysregulation, SD‐ILD may involve alveolar epithelial type II (AECII) cell injury and aberrant activation of lung‐resident fibroblasts [21, 22]. AECII injury impairs surfactant production and epithelial repair, while activated myofibroblasts drive extracellular matrix deposition and tissue stiffening [21]. Lung‐resident macrophages may further contribute to the maintenance of local inflammatory responses [23]. These lung‐specific elements may provide the structural context within which IFN‐γ‐associated signaling networks contribute to disease progression.

Serum biomarkers such as Krebs von den Lungen‐6 (KL‐6) and surfactant protein D (SP‐D) have been associated with the presence and severity of interstitial lung involvement in connective tissue disease‐associated ILD [24]. More recently, a prospective multicenter cohort study of 395 patients with SD‐ILD identified higher serum CA125 levels as independently associated with adverse outcomes, including death or lung transplantation [25]. IFN‐γ‐responsive chemokines, including CXCL9 and CXCL10, show altered expression patterns in the serum and salivary gland tissues of patients with SD, supporting their involvement in systemic and tissue‐level immune activation [26].

## 4 IFN‐γ: Fundamental Features and Pleiotropic Functions

IFN‐γ is classified as a type II interferon and is structurally and functionally distinct from both type I and type III interferons [27]. The cytokine circulates as a homodimer and signals through the high‐affinity IFN‐γ receptor (IFNGR), a tetrameric complex composed of two IFNGR1 (α) and two IFNGR2 (β) chains that is widely displayed on both immune and non‐immune cells [28]. IFN‐γ is produced chiefly by activated CD4^+^ Th1 cells, CD8^+^ T cells, and NK cells [29, 30, 31]. Additional cellular sources have also been reported to contribute to IFN‐γ production. These include KLRB1 + CD4 + T cells, mucosal‐associated invariant T (MAIT) cells, B cells, innate lymphoid cells (ILCs), and antigen‐presenting cells (APCs) [32, 33]. Upon engagement of its heterotetrameric receptor complex, IFN‐γ launches a transcriptional program that touches virtually every arm of immunity. Its portfolio spans immunomodulation, antiviral defense, tumor surveillance, and the orchestration of inflammatory responses [34, 35]. IFN‐γ serves as the principal macrophage‐activating factor, markedly enhancing macrophage phagocytic capacity, antigen‐presentation efficiency, and microbicidal activity [36]. IFN‐γ also licenses dendritic cells by up‐regulating surface MHC‐I/II and co‐stimulatory molecules (e.g., CD80/CD86), thereby accelerating antigen presentation and steering naïve T cells toward a Th1 fate while concurrently suppressing Th2 polarization to preserve the Th1/Th2 equilibrium [37]. Within the inflammatory microenvironment, IFN‐γ not only transcriptionally primes a panel of pro‐inflammatory mediators—including TNF‐α, IL‐12, and IL‐1β—but also synergizes with TLR signals to intensify macrophage responsiveness to pathogen‐associated molecular patterns. This cooperation may contribute to a self‐reinforcing inflammatory loop that can sustain and amplify inflammatory responses [38]. Additionally, IFN‐γ can induce IRF1 expression, while IRF1 has been implicated in PANoptosis, an inflammatory form of programmed cell death [39]. Whether this mechanism operates in SD‐ILD remains to be determined.

## 5 Dysregulated IFN‐γ Expression in SD

IFN‐γ is markedly up‐regulated in Sjögren's disease and operates across multiple immunopathogenic circuits that contribute to disease activity [40]. An elevated Th1/Th2 ratio is significantly associated with SD‐ILD [41]. In SD, IFN‐γ‐associated immune activity has been observed in salivary gland tissue, with NK cells and T lymphocytes representing important cellular sources of IFN‐γ [42, 43]. Concurrently, CXCL9, CXCL10, and CXCL11 are sharply up‐regulated, potentially recruiting additional T cells that may reinforce inflammatory amplification [26, 44]. Histopathology reveals dense infiltrates of activated T cells within salivary and lacrimal glands of SD patients. Among these, Th1 cells secrete abundant IFN‐γ and TNF‐α, which may contribute to epithelial‐cell dysfunction, impaired glandular function, and B‐cell activation [45, 46]. Metabolic rewiring of CD4^+^T cells has emerged as a pathogenic driver in multiple autoimmune disorders, including rheumatoid arthritis and systemic lupus erythematosus [47, 48]. Genetic variation also shapes IFN‐γ output. Specifically, the rs2069705 polymorphism enhances STAT4 binding to the IFN‐γ promoter and is associated with increased susceptibility to Sjögren's disease [49]. IFN‐γ is further linked to a functional deficit of regulatory T cells (Treg); reduced Treg suppression may allow expansion of IFN‐γ‐producing Th1 cells and contribute to persistence of autoimmune responses [50]. In SD, both peripheral blood and salivary glands show reduced Treg numbers; the magnitude of this deficit inversely correlates with disease activity as quantified by ESSDAI [51]. Recent work shows that SD‐associated inflammation drives salivary‐gland epithelial‐cell (SGEC) death and consequent hypofunction, with IFN‐γ identified as a key pathogenic effector [52, 53, 54].

## 6 Type I Interferon Signaling in SD: A Comparative Perspective

It is important to contextualize IFN‐γ within the broader interferon landscape of SD. Type I interferons (IFN‐α/β), signaling via the JAK1/TYK2–STAT1/STAT2–IRF9 axis, are widely recognized as important contributors to SD pathogenesis, particularly in subsets with prominent glandular dysfunction and autoantibody positivity [28, 40, 55]. Transcriptomic analyzes of salivary glands frequently reveal a robust type I interferon signature that partially overlaps with, yet is distinct from, the IFN‐γ response [40]. While type I interferons primarily sustain plasmacytoid dendritic cell activation and B‐cell maturation, IFN‐γ is strongly associated with Th1 polarization and macrophage activation [40, 55]. The relative and potentially cooperative roles of type I and type II interferon signaling in SD‐ILD therefore warrant further investigation.

## 7 IFN‐γ‐associated Signaling Networks Linking Inflammation to Fibrosis Progression in SD‐ILD

In the conceptual framework proposed in this review, IFN‐γ is considered a potential signaling node within an interconnected network involving the JAK/STAT, PI3K/AKT, NF‐κB, MAPK, WNT, and IDO–Kyn–AhR pathways. Importantly, the evidence supporting these pathways is heterogeneous. Direct mechanistic evidence in SD‐ILD is limited, whereas additional support is derived from studies in Sjögren's disease without characterized ILD, related connective tissue disease‐associated ILDs, fibrotic lung diseases, and experimental models. These different evidence levels are explicitly distinguished throughout the following sections. These pathways may collectively contribute to the connection between systemic immune dysregulation and sustained pulmonary inflammation and fibrotic remodeling. However, the extent to which IFN‐γ organizes or regulates this network specifically in SD‐ILD remains incompletely established. Accordingly, the proposed relationships should be regarded as a conceptual mechanistic model rather than a fully validated signaling hierarchy in SD‐ILD (Figure 1).

**Figure 1:**
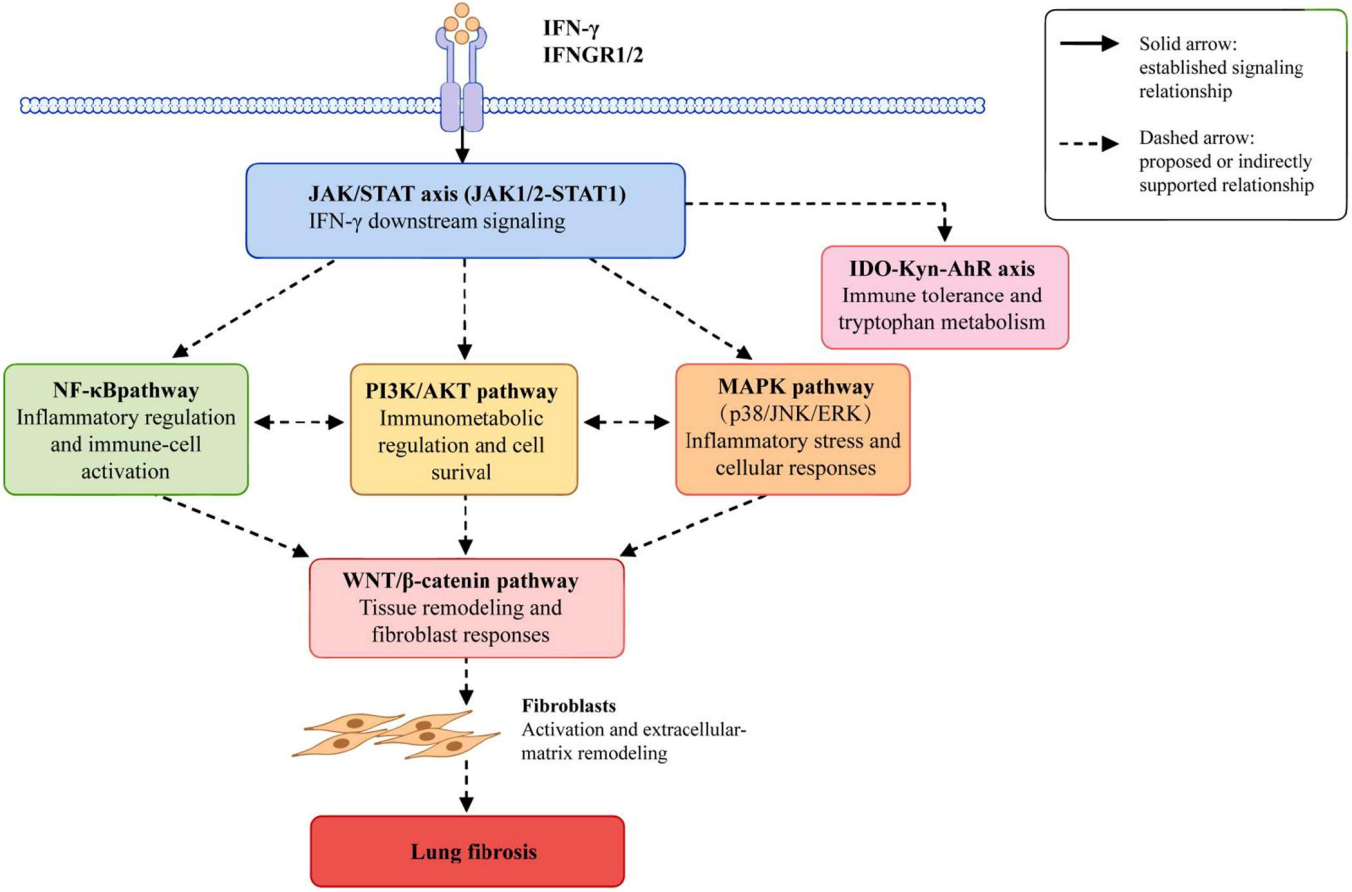
Proposed IFN‐γ‐associated signaling network potentially linking inflammation to fibrotic remodeling in SD‐ILD.

### 7.1 IFN‐γ–JAK/STAT Axis: A Potential Inflammatory Signaling Module in SD‐ILD

IFN‐γ exerts its immunoregulatory effects principally through the JAK/STAT pathway, with STAT1 phosphorylation mediated by JAK1/JAK2 representing the established downstream event [55, 56]. Accordingly, IFN‐γ–JAK/STAT signaling may provide one mechanistic link between systemic immune dysregulation and pulmonary inflammation in SD‐ILD. Persistent activation of this axis could contribute to sustained inflammatory signaling and subsequent tissue remodeling [57, 58].

Macrophages are well‐established cellular responders to IFN‐γ–JAK/STAT signaling. IFN‐γ can promote STAT1‐dependent transcriptional programs associated with macrophage activation, enhanced antigen‐presentation capacity, and increased inflammatory mediator production [10]. Their specific contribution to macrophage activation in SD‐ILD, however, remains to be directly demonstrated [59]. Experimental studies further indicate that IFN‐γ can promote macrophage polarization toward a pro‐inflammatory phenotype, providing a potential mechanism through which macrophages may contribute to the persistence of inflammatory responses [44].

CXCR3 ligands, particularly CXCL9 and CXCL10, are interferon‐responsive chemokines involved in the regulation of immune‐cell responses. In other inflammatory lung conditions, increased CXCL9/CXCL10 signaling has been associated with enhanced T‐cell responses and CXCR3 expression [45]. A comparable IFN‐γ–JAK/STAT–CXCR3 chemokine axis may contribute to immune‐cell recruitment and persistence in SD‐ILD [48] (Figure 2).

**Figure 2:**
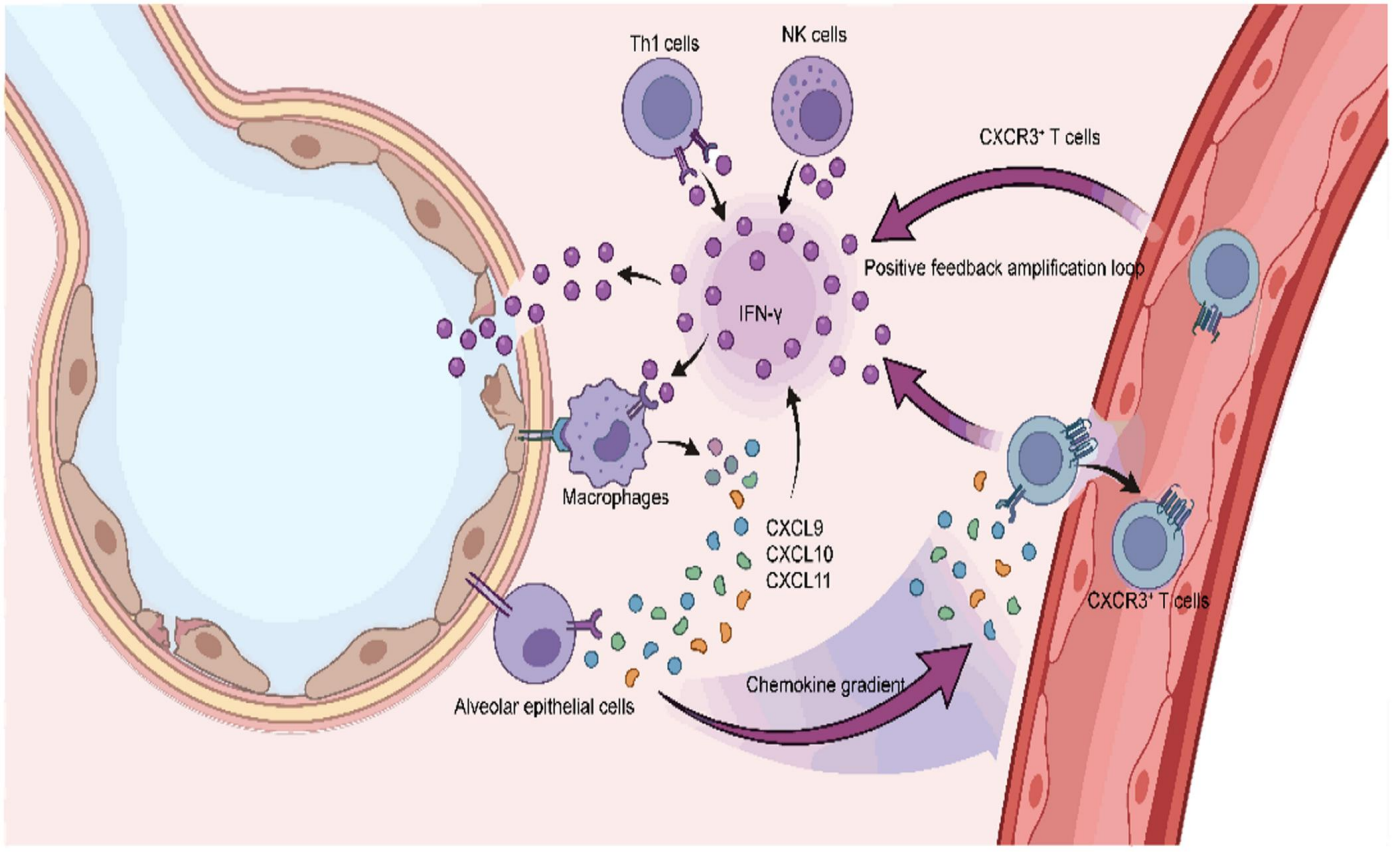
Proposed IFN‐γ‐associated immune amplification and chemokine‐mediated T‐cell recruitment in SD‐ILD.

At the network level, the IFN‐γ–JAK/STAT pathway engages in extensive cross‐talk with NF‐κB, PI3K/AKT, and MAPK cascades, creating a multi‐axis signaling web that amplifies and sustains the inflammatory output [13]. In other inflammatory disease models, STAT1‐dependent transcriptional programs can interact with additional cytokine‐driven pathways to reinforce inflammatory cellular states [60, 61]. Such cross‐talk may provide a plausible mechanism for sustained inflammatory signaling in SD‐ILD.

Collectively, available evidence supports the biological plausibility of IFN‐γ–JAK/STAT signaling as one component of the inflammatory network potentially relevant to SD‐ILD. Through its effects on immune‐cell activation and interactions with other inflammatory pathways, persistent JAK/STAT signaling may contribute to disease chronicity and subsequent fibrotic remodeling.

## 8 IFN‐γ–PI3K/AKT Axis: A Potential Immunometabolic and Immune‐Amplification Pathway

The PI3K/AKT cascade governs cell survival, metabolism, and differentiation, serving as a critical signaling hub for immune‐cell homeostasis and autoimmune responses [62]. Although PI3K/AKT is not considered a canonical direct downstream pathway of IFN‐γ signaling, interactions between IFN‐γ‐associated inflammatory responses and PI3K/AKT signaling have been described in other immunological contexts, suggesting a potential mechanism by which this pathway could contribute to immune‐cell activation and inflammatory amplification [63]. Direct evidence linking IFN‐γ to PI3K/AKT activation in SD‐ILD is currently lacking and requires disease‐specific validation.

IFN‐γ can alter the activation state of human monocytes and modulate their inflammatory phenotype [64]. More broadly, PI3K/AKT signaling is closely linked to metabolic and survival programs in immune cells, while metabolic remodeling is increasingly recognized as an important feature of chronic inflammation [62]. Together, these observations support a potential interaction between IFN‐γ‐associated inflammation and PI3K/AKT‐dependent immunometabolic responses. PI3K/AKT signaling is an important regulator of B‐cell survival, activation, and differentiation. Studies in systemic autoimmune diseases, particularly lupus, have demonstrated interactions between IFN‐γ‐related inflammatory signals and PI3K‐dependent B‐cell responses [65]. A similar IFN‐γ–PI3K/AKT‐mediated B‐cell program may contribute to immune dysregulation in SD‐ILD. In SD, aberrant B‐cell activation and autoantibody production represent well‐recognized features of disease pathogenesis [66, 67]. In connective tissue disease‐associated ILDs, persistent humoral immune activation has been proposed to contribute to pulmonary inflammation and tissue injury [22].

At the network level, PI3K/AKT signaling can interact with JAK/STAT, NF‐κB, and MAPK pathways, forming an interconnected regulatory network that may amplify inflammatory responses under chronic immune stimulation [68]. AKT signaling can interact with inflammatory transcriptional and metabolic programs, including mechanisms involving IKK activity, thereby potentially promoting immune‐cell survival and sustained responsiveness under chronic inflammatory conditions [69]. Such multi‐pathway interactions may contribute to persistent inflammatory signaling and influence subsequent tissue remodeling under chronic immune stimulation.

Accordingly, the IFN‐γ–PI3K/AKT axis may represent an amplification module linking inflammatory signaling, immunometabolic regulation, and B‐cell responses.

## 9 IFN‐γ–NF‐κB Axis: A Potential Regulator of Chronic Inflammatory Responses in SD‐ILD

The NF‐κB pathway is a key regulator of inflammation and immune‐cell survival that integrates multiple inflammatory signals and may contribute to disease chronicity across autoimmune disorders [13]. Direct evidence of IFN‐γ‐driven NF‐κB amplification in SD‐ILD remains limited, with mechanistic support derived primarily from other chronic inflammatory disease models. In other chronic inflammatory conditions, IFN‐γ can cooperate with additional inflammatory stimuli to enhance NF‐κB‐associated responses, including in alveolar macrophages and epithelial cells [70, 71], suggesting a potential mechanism for pulmonary inflammatory amplification in SD‐ILD. In monocytes and macrophages, IFN‐γ can modulate cellular responsiveness to additional inflammatory signals through cross‐regulation with other signaling pathways, including NF‐κB‐associated mechanisms [72]. Such interactions may contribute to the persistence of a pro‐inflammatory macrophage state in SD‐ILD.

Within the T‐cell compartment, NF‐κB signaling contributes to the regulation of immune‐cell activation, survival, and inflammatory responses [73]. IFN‐γ‐associated cytokine networks may influence T‐cell activity through interactions with multiple signaling pathways, potentially contributing to a T‐cell–macrophage inflammatory amplification loop in SD‐ILD.

At the signaling‐network level, NF‐κB interacts with multiple inflammatory pathways, including JAK/STAT and PI3K/AKT signaling, forming a broader regulatory network involved in immune‐cell activation and inflammatory regulation [13].

### 6.4 IFN‐γ–MAPK Axis: A Potential Regulator of Inflammatory Stress Responses and Fibrotic Remodeling

The MAPK cascade serves as an integrative signaling pathway that coordinates inflammatory signals, stress signals, and growth‐factor input to regulate cellular responses [74]. In fibrotic lung disease, it may function as an integrative pathway for multiple inflammatory signals and potentially contribute to disease progression [21]. Direct longitudinal or interventional studies of the IFN‐γ–MAPK axis in SD‐ILD are not yet available, with current mechanistic support derived from other chronic inflammatory and fibrotic lung disease models. In chronic inflammatory and fibrotic disease models, MAPK signaling has been implicated in inflammatory amplification and tissue‐remodeling processes [62, 75], suggesting that IFN‐γ‐associated MAPK activation may contribute to the transition from inflammation to fibrotic remodeling in SD‐ILD.

In monocytes and macrophages, IFN‐γ can modulate responses to inflammatory stimuli through interactions with TLR‐related signaling networks and downstream inflammatory pathways [76]. In pulmonary epithelial models, MAPK signaling has been shown to regulate inflammatory mediator expression, including IL‐8 production, suggesting a potential role in sustaining inflammatory responses in chronic lung conditions [77]. These findings raise the possibility that similar MAPK‐dependent mechanisms may contribute to macrophage or epithelial‐cell dysfunction in SD‐ILD.

In alveolar epithelial cells, MAPK signaling can integrate cellular stress and inflammatory stimuli to regulate injury responses and phenotypic changes. Experimental studies have shown that MAPK activation contributes to epithelial‐cell injury under mechanical stress conditions [78]. In other inflammatory diseases, IFN‐γ‐associated p38/ERK activation has been linked to epithelial‐to‐mesenchymal transition, suggesting a potential connection between inflammatory signaling and pro‐fibrotic epithelial responses [79].

At the disease‐progression level, chronic MAPK activation may promote the production of multiple pro‐fibrotic mediators and interact with IFN‐γ‐associated JAK/STAT and NF‐κB pathways, potentially linking persistent inflammatory signals with fibroblast activation and tissue‐remodeling programs [55, 75]. Thus, across diverse chronic‐inflammation models, MAPK signaling may function as an integrator of inflammatory stress and cellular responses, linking persistent inflammation with fibrotic remodeling [75]. The relevance of this IFN‐γ–MAPK interaction to inflammation‐to‐fibrosis progression in SD‐ILD requires direct validation.

### 9.1 IFN‐γ–WNT Axis: A Potential Regulator of Fibroblast Activation and Fibrotic Remodeling

The WNT signaling cascade is a well‐established pathway governing cell proliferation, differentiation, and tissue development; in fibrotic disorders it has emerged as a key driver of pathological remodeling. Recent studies show that chronic inflammation can switch WNT from a developmental signal to a profibrotic remodeling program [80]. In SD and SD‐ILD, IFN‐γ is not considered a direct activator of WNT signaling; rather, it may influence WNT pathway activity indirectly through inflammatory‐cell and structural‐cell interactions. Altered WNT/β‐catenin signaling has been documented in SD salivary glands [81], whereas its role in SD‐ILD lung tissue remains poorly characterized. Evidence from IPF and other fibrotic lung diseases nevertheless supports the biological plausibility of WNT/β‐catenin signaling as a potential contributor to pulmonary fibrotic remodeling.

Across chronic lung diseases, WNT/β‐catenin components are consistently up‐regulated in lung tissue, and the magnitude of activation correlates tightly with fibroblast activation, collagen deposition, and tissue stiffness [82]. Together, these observations support the biological plausibility of WNT/β‐catenin signaling as a contributor to pulmonary fibrotic remodeling relevant to SD‐ILD.

Within a chronic inflammatory niche influenced by IFN‐γ signaling, macrophages, T cells, and injured alveolar epithelium may release TNF‐α, IL‐1β, and TGF‐β, contributing to a cytokine microenvironment associated with persistent tissue activation. These mediators have been reported to influence β‐catenin stability and nuclear translocation in fibrotic models. Chronic inflammatory stress concurrently represses multiple WNT antagonists, lifting negative restraint and allowing the pathway to shift from a transient repair signal to a persistent, pathological remodeling program [83].

Fibroblasts are important cellular responders to WNT signaling. In fibrotic disease models, aberrant WNT activation has been associated with fibroblast proliferation, myofibroblast differentiation, and increased extracellular‐matrix production [80]. Sustained β‐catenin activity may promote fibroblast activation and persistence, while interactions between WNT and TGF‐β signaling may further enhance profibrotic responses. Both canonical and non‐canonical WNT signaling may promote fibroblast activation and sustained extracellular‐matrix remodeling through effects on proliferation, differentiation, migration, and cytoskeletal dynamics [84]. Accordingly, WNT signaling may represent a downstream remodeling pathway through which persistent inflammatory signals are linked to fibroblast activation and structural remodeling. Its specific contribution to fibrotic progression in SD‐ILD, however, remains to be established.

### 9.2 IDO–Kyn–AhR Axis: A Potential Regulator of Immune Adaptation and Fibrotic Remodeling

Direct evidence for the IDO–Kyn–AhR axis in SD‐ILD is currently lacking, and its inclusion here is based on mechanistic evidence from other chronic inflammatory and fibrotic disease contexts. Under persistent IFN‐γ exposure, IDO expression can be induced in monocyte–macrophages and dendritic cells, promoting tryptophan metabolism toward kynurenine production and subsequent AhR activation. This pathway has been associated with suppression of effector T‐cell responses and regulation of immune tolerance in several inflammatory contexts [85]. In chronic disease models, prolonged activation of this pathway may reshape local immune responses and cellular interactions. The IDO–Kyn–AhR axis may therefore influence immune regulation and immune–structural cell interactions within chronic inflammatory and fibrotic environments [85, 86]. Accordingly, its relevance to SD‐ILD should be regarded as exploratory and hypothesis‐generating rather than as an established disease‐specific mechanism.

## 10 Discussion and Perspectives

Collectively, the evidence synthesized in this review supports a conceptual model in which IFN‐γ may function in SD‐ILD not as an isolated pro‐inflammatory cytokine, but as a potential regulatory node within an interconnected pathogenic network.

The proposed organization of upstream inflammatory signals, intermediate amplification pathways, and downstream fibrotic remodeling processes represents a conceptual framework synthesized from pathway relationships observed across chronic inflammatory and fibrotic conditions. This framework is intended to guide hypothesis generation and therapeutic reasoning rather than to assert a fully validated biological sequence specific to SD‐ILD. Prospective validation in longitudinal SD‐ILD cohorts, ideally integrating spatial transcriptomics and single‐cell profiling, will be required to confirm the temporal sequence and relative contribution of individual pathways.

Within this framework, IFN‐γ may link systemic immune dysregulation to sustained pulmonary inflammation and, potentially, fibrotic remodeling through interactions with JAK/STAT, PI3K/AKT, NF‐κB, MAPK, WNT, and IDO–Kyn–AhR signaling. Rather than operating through a single linear cascade, IFN‐γ may participate in a multilayered signaling network in which upstream immune activation, intermediate amplification and maintenance modules, and downstream effector programs may collectively contribute to disease progression.

At the upstream level, the IFN‐γ–JAK/STAT axis may represent an important inflammatory signaling pathway that can influence activation states of monocytes, macrophages, and T cells. Chronic STAT1 activation may enhance antigen presentation and chemokine production, thereby potentially promoting immune‐cell recruitment and persistence within lung tissue in SD‐ILD. Importantly, cross‐talk between JAK/STAT and NF‐κB or PI3K/AKT signaling may contribute to the maintenance and amplification of inflammatory responses under chronic immune stimulation. This network‐level amplification may provide a mechanistic framework for understanding persistent inflammatory responses in SD‐ILD.

At the intermediate level, PI3K/AKT, NF‐κB, and MAPK pathways may function primarily as amplification and regulatory modules within the proposed IFN‐γ‐associated network rather than as independent disease initiators. Through effects on immunometabolic regulation, cell survival, and responsiveness to inflammatory signals, these pathways may contribute to sustained immune‐cell activation and prolonged inflammatory responses. In this context, MAPK signaling may represent an important integrator of inflammatory stress, linking immune‐derived signals to epithelial responses, repair processes, and epithelial–mesenchymal plasticity. Evidence from related autoimmune and fibrotic lung diseases supports the biological plausibility of MAPK signaling as a potential link between chronic inflammation and fibrotic remodeling within this framework.

Following persistent inflammation, fibrotic responses may become increasingly reinforced through interactions among immune, stromal, and tissue‐remodeling pathways. WNT/β‐catenin signaling may act as a fibroblast‐associated remodeling pathway that links inflammatory signals with structural changes. Aberrant WNT activation has been associated with fibroblast proliferation, myofibroblast differentiation, extracellular‐matrix deposition, and altered cellular persistence, which may contribute to sustained extracellular‐matrix remodeling in fibrotic conditions. Within this framework, fibrotic remodeling in SD‐ILD may become progressively less dependent on the initiating inflammatory signals as stromal and extracellular‐matrix remodeling pathways become established.

More speculatively, chronic IFN‐γ exposure may influence the IDO–Kyn–AhR axis, introducing a potential layer of immune modulation. In other chronic inflammatory settings, its effects on effector T‐cell responses and immune–stromal interactions provide a hypothesis for how this axis might connect immune regulation with fibrotic remodeling.

From a therapeutic perspective, this network‐based model may help identify pathways for future investigation. Among these pathways, the relatively well‐characterized JAK/STAT axis may represent a more immediate candidate for investigating modulation of upstream immune activation and inflammatory amplification. However, the extensive cross‐talk among downstream pathways suggests that single‐pathway inhibition may be insufficient to address the complexity of established fibrotic disease. The extensive pathway cross‐talk identified in this framework provides a conceptual rationale for investigating combinatorial approaches that address both inflammatory and fibrotic components of disease. These considerations underscore the importance of stage‐specific and pathway‐informed therapeutic approaches.

### 10.1 Evidence Grading

To enhance methodological rigor, we assessed the strength of evidence supporting the proposed signaling network using a qualitative evidence‐strength framework adapted for mechanistic reviews.

Relatively stronger evidence: The biological relevance of the IFN‐γ–JAK/STAT axis is supported by evidence of IFN‐γ‐associated immune activation in SD, mechanistic studies supporting STAT1‐dependent inflammatory and chemokine responses, and broader mechanistic evidence from related autoimmune and inflammatory conditions [53, 54, 66]. However, direct evidence establishing the activity and therapeutic relevance of this axis specifically in SD‐ILD remains limited.

Intermediate‐level evidence: The involvement of PI3K/AKT, NF‐κB, and MAPK pathways is supported by mechanistic studies demonstrating their roles in inflammatory signaling, immune‐cell regulation, and pathway cross‐talk, together with concordant findings from related autoimmune, inflammatory, and fibrotic disease models. However, direct mechanistic and interventional evidence for these pathways in SD‐ILD remains limited.

Lower‐level evidence: The potential relevance of WNT/β‐catenin signaling to SD‐ILD is supported by evidence from SD‐related tissues and mechanistic studies in idiopathic pulmonary fibrosis and other fibrotic lung diseases. Although the profibrotic role of WNT signaling is well established across fibrotic contexts, its disease‐specific contribution to SD‐ILD remains insufficiently characterized.

Exploratory‐level evidence: The relevance of the IDO–Kyn–AhR axis to SD‐ILD remains hypothesis‐generating and is supported primarily by mechanistic evidence from other chronic inflammatory and fibrotic disease contexts. In addition, the hierarchical organization into “upstream inflammatory signals,” “intermediate amplification pathways,” and “downstream fibrotic remodeling processes” represents a theoretical synthesis requiring prospective validation.

Implications: The relatively stronger mechanistic evidence supporting JAK/STAT signaling provides a rationale for further investigation of JAK/STAT‐targeted interventions in SD‐ILD. Pathways supported by intermediate‐ or lower‐level evidence represent rational but investigational targets. Fibrosis‐related pathways represent investigational therapeutic targets whose relevance should be evaluated according to disease stage and SD‐ILD‐specific evidence.

Several limitations should be acknowledged. Much of the mechanistic framework proposed here is derived from associative data, experimental models, or extrapolation from related autoimmune and fibrotic lung diseases. Direct longitudinal and interventional studies specifically focused on SD‐ILD remain scarce, particularly with respect to defining the temporal sequence and relative contribution of individual signaling pathways. Moreover, disease heterogeneity—including variability in immune‐cell composition, inflammatory burden, and genetic background—likely influences the relative contributions of specific pathways across patients and disease stages.

In summary, this review provides an integrated perspective on SD‐ILD by proposing a network‐based model in which IFN‐γ may function not merely as an inflammatory mediator, but as a potential regulatory node within a network involving immune activation, inflammatory amplification, metabolic reprogramming, and fibrotic remodeling. The present synthesis integrates JAK/STAT, PI3K/AKT, NF‐κB, MAPK, WNT, and IDO–Kyn–AhR signaling into a unified mechanistic framework. This integrative perspective emphasizes a shift from linear inflammatory cascades toward multidimensional signaling networks that may better reflect disease complexity.

From a translational perspective, this framework provides a basis for hypothesis‐driven therapeutic investigation. By distinguishing upstream inflammatory signals from downstream fibrotic remodeling processes, this framework may provide a conceptual rationale for stage‐specific therapeutic investigation and highlight potential combinatorial treatment strategies. The model further highlights the potential value of investigating pathway‐informed biomarkers for patient stratification and disease monitoring. If prospectively validated, this network‐based framework may ultimately contribute to more precise and individualized management of patients with SD‐ILD.

Future research integrating longitudinal clinical cohorts with spatial transcriptomics and single‐cell profiling will be critical to clarify the relative contributions of individual pathways across disease stages and refine therapeutic targeting strategies.

### 10.2 Therapeutic Implications

The proposed signaling network may provide a conceptual framework for considering potential therapeutic strategies. JAK inhibitors (e.g., tofacitinib, baricitinib, and upadacitinib) represent a mechanistically plausible approach for modulating JAK/STAT signaling and may therefore influence IFN‐γ‐associated inflammatory responses [56, 68]. Evidence from related autoimmune and inflammatory conditions provides a rationale for investigating JAK inhibition in SD‐ILD, particularly in inflammation‐dominant disease, although its clinical benefit in SD‐ILD remains to be established.

B‐cell–targeted therapies represent another therapeutic approach that may intersect with IFN‐γ‐associated immune networks. Agents such as rituximab and belimumab target distinct components of B‐cell biology and therefore provide a conceptual basis for investigating B‐cell–directed strategies within the broader immune network relevant to SD. Although neither agent has been specifically evaluated in SD‐ILD phase III trials, B‐cell–targeted therapy may warrant further investigation in SD‐ILD. Recent real‐world evidence from a multicenter European cohort of 191 patients with SD‐ILD showed that rituximab was used in 25.1% of patients, providing direct evidence of its use in contemporary clinical practice, although treatment efficacy cannot be established from these observational data [87].

In the setting of established fibrotic remodeling, therapeutic strategies may need to consider both inflammatory and fibrosis‐associated mechanisms. Among antifibrotic agents used in fibrotic lung disease, nintedanib has demonstrated efficacy in patients with progressive fibrosing ILDs across a range of underlying diagnoses [88]. Within the proposed IFN‐γ‐associated network, WNT/β‐catenin and MAPK signaling are among the pathways implicated in fibrotic remodeling [21, 84]; however, nintedanib does not directly target IFN‐γ signaling. These findings provide a rationale for investigating antifibrotic therapy in SD‐ILD patients who develop a progressive fibrotic phenotype.

PI3K/AKT and MAPK remain investigational therapeutic targets in the context of SD‐ILD. Although pathway‐targeting approaches have shown potential in preclinical or mechanistic studies across autoimmune and inflammatory disease models [74, 89], their therapeutic relevance to SD‐ILD has not been established.

Collectively, these considerations provide a conceptual rationale for investigating stage‐specific therapeutic strategies, including immune‐directed approaches in inflammation‐dominant disease and antifibrotic therapy in progressive fibrotic disease. Combination strategies and pathway‐specific small‐molecule inhibitors remain longer‐term investigational possibilities. Biomarkers—including serum CXCL9/CXCL10 and KL‐6—may warrant investigation as potential tools for patient stratification and disease monitoring [24, 26].

## Author Contributions

**Qianqian Chen:** conceptualization, methodology, data curation, investigation, validation, formal analysis, visualization, writing – original draft. **Yuhan Li:** software, formal analysis, writing – review and editing. **Yiming Chen:** data curation, visualization. **Yuhua Su:** investigation, visualization, writing – review and editing. **Xiaodong Wang:** conceptualization, supervision, project administration, writing – review and editing. **Chunxiao Guan:** conceptualization, methodology, validation, supervision, writing – review and editing.

## Data Availability

Data sharing not applicable to this article as no datasets were generated or analyzed during the current study.
